# Mental health help-seeking preferences and behaviour in elite male rugby players

**DOI:** 10.1136/bmjsem-2023-001586

**Published:** 2023-05-26

**Authors:** Saki Oguro, Yasutaka Ojio, Asami Matsunaga, Takuma Shiozawa, Shin Kawamura, Goro Yoshitani, Masanori Horiguchi, Chiyo Fujii

**Affiliations:** 1 Department of Community Mental Health and Law, National Center of Neurology and Psychiatry, Kodaira, Tokyo, Japan; 2 Department of Mental Health and Psychiatric Nursing, Tokyo Medical and Dental University, Bunkyo-ku, Japan; 3 Japan Rugby Players Association, Tokyo, Japan

**Keywords:** rugby, athlete, mental, psychology

## Abstract

**Objectives:**

Mental health symptoms and mental illnesses are common in elite athletes. There is an urgent need to develop care systems to support the mental health of elite athletes. Understanding elite athletes’ preferences in mental health help seeking can help explore strategies to develop such systems. Therefore, this study aims to investigate with whom/where elite athletes feel comfortable discussing mental health concerns and seeking help.

**Methods:**

We analyse data from 219 Japanese male rugby players out of 612 players (565 Japanese, 47 foreigners) aged 18 and over who belong to the Japan Rugby Players Association using a cross-sectional design and an anonymous, web-based, self-administered questionnaire. In the questionnaire, the players are asked to rate on a 5-point Likert scale how comfortable they feel talking about their mental health concerns with affiliation/team staff, family/relatives, friends, mental health professionals, rugby-related seniors and teammates. Analysis of variance and Dunnett’s test are performed to detect differences in their preferences for sources of help.

**Results:**

Dunnett’s test shows that the mean scores for preferring to consult affiliation/team staff are significantly lower than for all the other groups (p<0.001), indicating that players are reluctant to seek help for mental health concerns from affiliation/team staff. Fewer players sought help from affiliation/team staff or mental health professionals than from other groups.

**Conclusion:**

Regarding mental health concerns, for elite male rugby players as elite athletes, it can be difficult to ask for help or talk to team staff.

**WHAT IS ALREADY KNOWN ON THIS TOPIC**

- Understanding the mental health help seeking of elite athletes can inform the development of effective mental healthcare systems in a highly competitive sports society. However, the details of help-seeking preferences and actual help-seeking behaviours of elite athletes are unclear.

**WHAT THIS STUDY ADDS**

- Our study suggests that elite athletes may struggle to talk to team members and staff about mental health issues and may be less likely to seek help.

**HOW THIS STUDY MIGHT AFFECT RESEARCH, PRACTICE OR POLICY**

- The highly competitive sports society demands the creation of a psychologically safe environment where elite athletes can discuss their mental health concerns.

## Background

There has been growing interest in mental health needs in highly competitive sports environments and the development of an effective mental healthcare system for elite athletes. Through a systematic review by the IOC Mental Health Consensus Panel,^1^ a consensus statement on mental health-related practice for the high-performance community, including elite athletes, team staff and management, has been published based on international academic findings. Other international sports organisations and academic societies have also published statements on the mental health of elite athletes.^2–9^ Statements and review papers have explained that mental health symptoms are common in elite athletes.^1–10^ A previous meta-analysis by Gouttebarge *et al* found that 33.6% of elite athletes experienced mental health symptoms, including depression and anxiety.^11^ It has also been reported that there is a higher prevalence of mental illnesses, such as eating disorders, in elite athletes than in general populations.^12 13^ In Japan, our previous research has also shown that Japanese male rugby players, as elite athletes, also experience mental health symptoms at a prevalence similar to that of elite athletes of other nationalities.^14 15^ Identified risk factors for such mental health symptoms include factors shared with the general population of young people (traumatic events, life stressors, relationship difficulties, financial hardship, etc) and factors specific to elite athletes (injury, selection, retirement, travel, media exposure, etc).^1 16 17^ In addition, previous papers^18 19^ have suggested an association between the experience of concussion and impaired mental health, particularly depression, underlying possible neurobiological conditions, such as chronic traumatic encephalopathy.^19^

Mental health help seeking in elite athletes is recognised as an important issue.^1–10 20 21^ Delaying mental healthcare for mental health symptoms can lead to poor athletic performance and even retirement from competition in severe cases.^22 23^ However, in the current situation, elite athletes are often reluctant to seek help from others.^20 21 24^ A lack of mental health knowledge is considered one of the main barriers preventing elite athletes from seeking help.^20 21 25^ Recently, we found that elite athletes who need mental health support tend not to ask others for help, even if they have greater knowledge,^26^ suggesting that a psychologically safe environment that allows for help seeking is required.^24 26 27^ Mental health-related stigma, like low mental health literacy, is also a common barrier to elite athletes seeking mental health treatment.^20 21^

Understanding the preferences of elite athletes for mental health help seeking can inform the development of effective mental healthcare systems in a highly competitive sports society. Following an IOC survey about help seeking in sports settings, it was reported that the respondents (n=360; active and former elite athletes, coaches and representatives of sports governing bodies) would approach friends and fellow athletes (50%), family (40%) and coaches (8%) when experiencing mental health symptoms.^28^ Another paper showed that 54% of coaches would prefer to receive mental health support from someone outside of their organisation, and only 7% of them favoured receiving support from someone inside their organisation.^29^ A study examining the help-seeking preferences of Japanese and American elite college athletes showed that Japanese college-level athletes were the least willing to seek help from their coaches.^30^ However, as far as we know, there are few studies, except for the IOC report in 2020,^28^ on the help-seeking preferences and actual help-seeking behaviour of elite athletes in the top categories, especially from whom/where they are most likely to seek help. Therefore, in the current study, we investigate and report the mental health help-seeking preferences and the actual behaviour of Japanese male rugby players as elite athletes.

## Methods

### Study design and setting

A cross-sectional survey design was implemented. Our report followed the Strengthening the Reporting of Observational Studies in Epidemiology guidelines for observational cross-sectional studies.^31^ In the survey process, the survey URL was distributed to all players belonging to the Japan Rugby Players Association (JRPA) through one or two team representatives who participated in regular meetings of the JRPA. The online survey was anonymous and took about 10 min to complete. The players were given an explanation of the survey process, the purpose of the study, data collection procedures and consent to participate on the web page. The players consented to participate and were provided with one-time access to the survey on a tablet or laptop computer using IP address filtering access. The data were collected shortly before the off-season from December 2020 to February 2021.

### Participants

We collected data from 612 rugby players (565 Japanese and 47 foreign players) aged 18 years or older registered with the JRPA. The current survey was available in both Japanese and English. Two hundred and twenty-seven of the 612 players completed the survey (response rate: 37.1%). The response rate for this survey was similar to, and no lower than, previous mental health surveys conducted in Japan.^32^ Of the 227 respondents, 219 who were born in Japan were analysed as the target group, as help-seeking characteristics and preferences may be influenced by cultural factors. All the investigators followed a learning course on research ethics.

### Measurements

We used the following two questions to examine the preferences for help seeking and the actual behaviour of players. The first question was about preferences for help seeking: ‘How comfortable would you feel talking about your mental health, for example, saying that you have mental health symptoms or illness and how this affects you?’ Each of the six groups, affiliation/team staff, family/relatives, friends, mental health professionals, rugby-related seniors (veterans and/or former players who are not teammates) and teammates, was rated on a Likert scale of 1–5, with higher scores indicating being easier to talk to. The second question was about the consultation experience: ‘In the last three months, have you consulted or received professional support for mental health concerns, including depression and anxiety?’ The players chose one of the two answers: ‘I did not experience any mental health problems’ or ‘I received mental health support’. The players who had received mental health support were asked to answer the next question: ‘Whom did you talk to?’ The players could select multiple answers from affiliation/team staff, family/relatives, friends, mental health professionals, rugby-related seniors, teammates and others.

### Background information/demographics

The players completed information and demographic survey items, including age, country of birth, educational attainments, marital status, the number (if any) of dependent children, residential status, experience on the national team and playing status for the last season.

### Statistical methods

For statistical data analysis, scores obtained from the first question were expressed as mean±SD for each group. All statistical analyses were performed with EZR (V.1.54),^33^ which is a graphical user interface for R. One-way analysis of variance (ANOVA) and Dunnett’s test were used to identify the significance between subgroups. P value <0.05 was considered significant. In the second question, we counted the number of people in each group whom elite athletes had consulted or from whom they had received support.

### Patient and public involvement

This research is a joint research with the JRPA, Japanese elite athletes, and a team was established to manage this research. As participants’ representatives, some of the elite athletes were consulted and provided input for the recruitment strategy. This working group also provided feedback on the structure and overall methodology of the larger study.

## Results

We analysed 219 players. Demographic characteristics are shown in table 1, with almost half of the players between 25 and 29 years old. Ninety-eight per cent had graduated from university, and one in five players had experience on the Japanese national team (table 1).

**Table 1:** Demographic characteristics of the study participants

	n (%)
Overall	219
Age	
20–24	61 (27.9)
25–29	100 (45.7)
30–34	47 (21.5)
35+	11 (5.0)
Educational level	
High school	2 (0.9)
Junior college or technical school	2 (0.9)
Four-year college or university	213 (97.3)
Postgraduate college (or higher)	2 (0.9)
Marital status	
Never married	113 (51.6)
Married	104 (47.5)
Divorced or widowed	2 (0.9)
Dependent children	
Yes	54 (24.7)
No	165 (75.3)
Residential status	
Living alone	48 (21.9)
Living with family and/or partner	105 (47.9)
Dormitory	66 (30.1)
Playing status of last season	
As an active member	75 (34.2)
As a reserve member	63 (28.8)
No play	81 (37.0)
Experience in the national team	
Yes	43 (19.6)
No	176 (80.4)

The mean scores of responses for the preferences regarding from whom to seek help were 2.90±1.33 (affiliation/team staff), 3.96±1.21 (family/relatives), 3.96±1.18 (friends), 3.63±1.11 (mental health professionals), 3.59±1.30 (rugby-related seniors) and 3.73±1.26 (teammates). The one-way ANOVA detected significant differences between groups (*F*(5, 1308=21.7, p<0.001)). Dunnett’s test, used to compare the mean scores for affiliation/team staff with those of the other groups, showed that the mean scores for affiliation/team staff were significantly lower than those of the other groups (table 2). Concerning actual help-seeking behaviour regarding mental health concerns, the number of elite athletes who had consulted or received support from them was lower for affiliation/team staff and mental health professionals (table 3).

**Table 2:** Comparison of the mean scores for seeking help from ‘Affiliation/team staff’ and other sources of help using Dunnett’s test

Sources of help	Mean difference	SE	95% CI	Dunnett’s test P value
a. Affiliation/team staff	Ref	Ref	Ref	a<b, c, d, e, f P<0.001
b. Family/relatives	1.05	0.12	(0.82, 1.29)	
c. Friends	1.05	0.12	(0.82, 1.29)	
d. Mental health professionals	0.73	0.12	(0.49, 0.96)	
e. Rugby-related seniors	0.69	0.12	(0.46, 0.92)	
f. Teammates	0.82	0.12	(0.59, 1.05)	

Ci, Confidence influence; Ref, reference; SE, Standard error .

**Table 3:** Number of players who sought help regarding mental health concerns in the last 3 months

Sources of help	Affiliation/team staff	Family/relatives	Friends	Mental health professionals	Rugby-related seniors	Teammates	Others
Players (n)	14	41	30	13	34	37	1

## Discussion

The current study presents the mental health help-seeking preferences and actual help-seeking behaviour in Japanese male rugby players. Our findings suggest that male elite athletes may be as reluctant to seek help from their team staff about mental health concerns as from mental health professionals, whom they normally have little opportunity to contact.

### Help-seeking preferences

We have reported the preferences regarding whom to consult for help with mental health concerns in elite athletes who are active in the top categories. Our results are consistent with previous findings in Japanese college athletes^30^ and active and former elite athletes,^28^ which showed that athletes were less likely to seek help for mental health concerns from coaches and team staff than from friends, family members or teammates. In the current highly competitive sports community, mental health symptoms and illness in elite athletes may be regarded as a lower priority than their physical health,^34^ and traditional stigma regarding mental health problems as a source of weakness or embarrassment may exist.^20 21 25^ Players may be concerned that if their team staff (team coach, etc) discover their mental health problems, their position on the team may be threatened, or they may ultimately lose their job.^29^ In the current high-performance sports environment, there is a strong demand for immediate performance. Thus, team staff, especially coaches, may influence elite athletes’ mental health coping strategies.^1^

### Actual help-seeking behaviour

The number of players who had sought help from team staff for mental health concerns was as few as those seeking help from mental health professionals. Given the general reluctance to seek help from mental health professionals,^35^ elite athletes find it difficult to seek help from the team staff. Based on this position, elite athletes are more likely to miss opportunities to talk about their mental ill health inside and outside the team and may become isolated if they have mental healthcare needs. Our previous research has shown that even elite athletes with greater mental health knowledge may feel unable to seek help when they experience poor mental health status.^26^ It is necessary to create a psychologically safe environment inside and outside of the team so that athletes can talk about themselves, including their mental health concerns.^24 27^

### Future challenges

To create a highly psychologically safe environment for elite athletes, it is required to improve the mental health literacy of elite athletes, the surrounding staff and the competitive community overall.^1 20 36 37^ Previous intervention research findings have shown that providing mental health literacy education can positively affect the mental health knowledge, attitudes, behavioural intentions and helping skills of elite athletes/surrounding staff.^36 37^ Several possible practical approaches have also been reported, including increasing peer teammates’ training in mental health knowledge and skills in mental health first aid, or the training of athletic trainers/physiotherapists in mental health recognition and their ability to make referrals to treatment.^38 39^ As a bridge to actual practice, the recently developed IOC Mental Health in Elite Athletes Toolkit is a mental health literacy educational resource in sports society and is expected to become a standard international tool.^40^ In addition, the implementation of an on-site care system in two pro sports leagues in the USA showed that the presence of on-site mental health providers resulted in increases in service utilisation.^41 42^ Besides this, the usefulness of longitudinal mental health screening (eg, preseason, after serious injury, expected or unexpected transition from sport) would further increase service utilisation.^28^ Building a support system outside the team, such as the Australian Institute of Sport mental health referral network, is also important.^43^ In the support system, elite athletes can contact the mental health referral network themselves and obtain free and confidential support or a referral to other sources of help, such as psychologists or psychiatrists.

### Strengths and limitations

This study is the first to quantify to whom elite athletes find it easier to talk and where they seek help regarding their mental health concerns. However, we should note the following limitations when interpreting the study results. First, the current samples were all from Japanese male rugby players. More research must be done to include female elite athletes and elite athletes from other sports, including individual sports. Second, the response rate was just below 40%, which is not as low as other mental health surveys in Japan,^30^ so it is possible that many of the participants had an interest in mental health. Third, we did not use a rigid measurement in assessing why the rugby players preferred/negated each group. Specific job descriptions of team staff should be clarified. For example, the participants may have taken team staff to refer to coaches, strength and conditioning staff, athletic trainers/physiotherapists, team doctor or team-based mental health providers. In addition, online self-help and consulting through the internet were not included in the selections for either preference or actual help. Recently, there have been expectations that web-based digital mental health support/care may potentially provide athletes with better accessibility.^44^ Moreover, such digital support tools may be available to record the need for psychological screening and tracking platforms in addition to mental health education to address the barriers to help seeking.^45^ Finally, we did not assess athletes who had not consulted anyone or received any support despite their experience of mental health issues.

## Conclusion

We have identified that it might be difficult for Japanese male rugby players as elite athletes to seek help from or talk to affiliation/team staff about mental health concerns. The results are consistent with the actual number of players who sought help. Our findings may indicate that players are currently in a position where they may feel unable to talk about themselves, including their mental health conditions. A psychologically safe environment is required both inside and outside the team. Affiliation/team staff may have a vital role to play in addressing barriers to help seeking to reduce stigma and foster psychological safety environments. We might also consider establishing a support system outside of the organisation.

## Data Availability

Data sharing not applicable as no data sets generated and/or analysed for this study. Data cannot be shared publicly because ethical restrictions exist. Publishing data sets is not covered by the informed consent given by the study participants, which the Research Ethics Committee of the National Center of Neurology and Psychiatry approved. The data are not owned by a third party.
